# Spirostanol Saponins from Flowers of *Allium Porrum* and Related Compounds Indicating Cytotoxic Activity and Affecting Nitric Oxide Production Inhibitory Effect in Peritoneal Macrophages

**DOI:** 10.3390/molecules26216533

**Published:** 2021-10-29

**Authors:** Juraj Harmatha, Miloš Buděšínský, Zdeněk Zídek, Eva Kmoníčková

**Affiliations:** 1 Institute of Organic Chemistry and Biochemistry, Czech Academy of Sciences, 166 10 Prague, Czech Republic; budesinsky@uochb.cas.cz; 2 Institute of Experimental Medicine, Czech Academy of Sciences, 142 20 Prague, Czech Republic; zdenek.zidek@iem.cas.cz (Z.Z.); eva.kmonickova@lfmotol.cuni.cz (E.K.); 3 Department of Pharmacology, Second Faculty of Medicine, Charles University, 150 00 Prague, Czech Republic

**Keywords:** *Allium porrum*, leek flowers, steroid saponins, aginoside, alliporin, cytotoxicity, NO production

## Abstract

Saponins, a diverse group of natural compounds, offer an interesting pool of derivatives with biomedical application. In this study, three structurally related spirostanol saponins were isolated and identified from the leek flowers of *Allium porrum* L. (garden leek). Two of them were identical with the already known leek plant constituents: aginoside (1) and 6-deoxyaginoside (2). The third one was identified as new component of *A. porrum*; however, it was found identical with yayoisaponin A (3) obtained earlier from a mutant of elephant garlic *Allium ampeloprasun* L. It is a derivative of the aginoside (1) with additional glucose in its glycosidic chain, identified by MS and NMR analysis as (2α, 3β, 6β, 25*R*)-2,6-dihydroxyspirostan-3-yl β-D-glucopyranosyl-(1 → 3)-β-D-glucopranosyl-(1 → 2)-[β-D-xylopyranosyl-(1 → 3)]-β-D-glucopyranosyl]-(1 → 4)-β-D-galactopyranoside, previously reported also under the name alliporin. The leek native saponins were tested together with other known and structurally related saponins (tomatonin and digitonin) and with their related aglycones (agigenin and diosgenin) for in vitro cytotoxicity and for effects on NO production in mouse peritoneal cells. The highest inhibitory effects were exhibited by 6-deoxyaginoside. The obtained toxicity data, however, closely correlated with the suppression of NO production. Therefore, an unambiguous linking of obtained bioactivities of saponins with their expected immunobiological properties remained uncertain.

## 1. Introduction

Spirostanol saponins belong to a large family of molecules composed of hydrophobic aglycones and hydrophilic sugar moieties and are remarkable for their versatile and significant biological effects as applied to ordinary food and feedstuffs [1], as well as to specific medical food supplements [2]. Equally important is their role in the ecological co-existence of insects and other organisms with plants that contain saponins [3]. Plants are not only natural sources of these substances but through their effects also a correlation factor of natural relationships between organisms. Their ecological role relates to their miscellaneous physiological activities and often depends on specific details in their chemical structure or on their quantitative content [3]. The physiological and pharmacological activity is, however, more extensive and depends intimately on the details of their chemical structure [1,2]. The wide structural variation of steroid saponins, especially those from the genus *Allium* [3,4,5,6], is reflected in various effects that range from beneficial to toxic [7], depending on a wide scale of various and complex biochemical and physiological mechanisms.

We focused our interest on the investigation of the immune response induced by a series of single isoprenoids. Initially, we tested phytoecdysteroids [8]; however, their immunobiological activity revealed only slight effects. Terpenoids proved to be more interesting, especially the significantly effective specific sesquiterpene lactones [9] and their different structurally modified derivatives [10,11].

**Figure 1:**
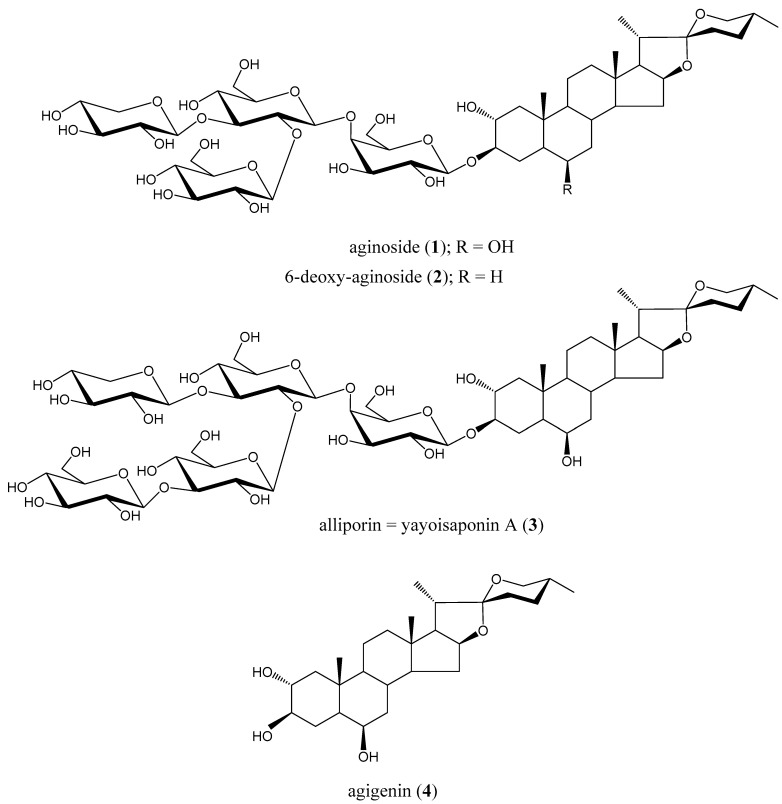
Structures of Compounds **1**–**4** isolated from *Allium porrum* flowers.

This range of tested structural types of isoprenoids is now complemented by selected spirostanol saponins from *Allium porrum,* some that were obtained during our earlier chemoecological studies [3,12,13]. Two previously known saponins, aginoside (**1**) and 6-deoxy-aginoside (**2**), were isolated together with what was at the time, a new, related saponin named alliporin (**3**) (see Figure 1). These three saponins were then tested to determine their chemoecological properties [14,15]. This paper expands our interest into the immunobiological activities of the leek flower saponins compared to the activities of various selected structurally related saponins alongside some relevant aglycones. We also present here our detailed structural analysis of the new leek flower constituent alliporin, confirming its identity with the previously reported yayoisaponin A (**3**), isolated earlier from the bulbs of an elephant garlic mutant [16]. Our analysis represents the most complete NMR characterization of saponins **1**–**3** obtained from leek flowers reported to date.

## 2. Results and Discussion

### 2.1. Chemical Identification and Structural Relations

Compounds **1**–**3** represent spirostanol type saponins, differing just by the number and/or position of hydroxy groups in their steroid part, as well as by the number or sequence of saccharides in their glycosidic part (Figure 1). The differences in their molecular structure became a subject of interest for investigating the relation between their structure and their immunobiological properties, as a continuation of our previous research [8,9,10,11].

In the beginning, we focused our interest only on the identification of the appropriate insect toxic compound located in leek flowers (*Allium porrum*) that inhibits the growth and development of leek-moth larvae (*Acrolepiopsis assectella*), which are highly specialized to feed on the leaves of various *Allium* plants [3,12]. The active insect toxic compound was isolated through accompanied simultaneous insect testing and was identified [13] as a spirostane type saponin aginoside (**1**), previously described as a constituent of *Allium giganteum* [17]. The proposed mechanism of toxicity related with the ecdysis disturbing effect was experimentally confirmed [14,15] and described [3]. At that time, only aginoside (**1**), the major saponin constituent was a subject of interest, because its activity was dependent primarily on its quantitative content [3,14]. Simultaneously, the two minor saponins **2** and **3** were also isolated and tested [15,18], but their structure was not fully elucidated at that time. For the present study, however, they are as important as the major aginoside (**1**), and we thus identified their structure. This was accomplished by NMR spectroscopic analysis, based on data comparison with results obtained during the structural identification of aginoside (**1**). It has been shown that their structures are related. The saponin **2** was identified as 6-deoxy-aginoside (**2**), formerly described as bulbs constituent of *Allium porrum* [19,20]. Saponin **3** was found to be a structurally related, only containing one extra sugar unit in the glycosidic moiety, then presented as alliporin [3,15]. Now it was proven to be structurally identical to yayoisaponin A (**3**) [16]. Besides the saponins **1**–**3**, one of their essential aglycone was also isolated in a small amount and identified as spirostan-2α, 3β, 6β–triol (**4**), already known and reported as agigenin [17].

The occurrence of four spirostane saponins in the leek bulbs was published earlier [19,20]. One of those saponins is identical to our 6-deoxyaginoside (**2**). The other three saponins are closely related derivatives of aginoside (**1**) and yayoisaponin A (**3**). The only difference is in the number and position of hydroxyls in their aglycone part. However, the occurrence of aginoside (**1**) was not reported in those papers. Nevertheless, in another paper [21], the same authors described the occurrence of agigenin (**4**) in *Allium porrum,* together with other structurally related sapogenins, as its hydroxy-, dehydro- or oxo- derivatives. Some of their glycosidic conjugates were later found in corresponding saponin fractions of *A. porrum* bulb extracts [22,23]. Surprisingly, a 6-deoxy derivative of yayoisaponin A was isolated from a related species *Allium rotundum* [24]. In addition, a 2-dehydro derivative was isolated from a mutant of *Allium ampeloprasum,* reported as yayoisaponin B [16]. Two other related saponins, structurally identified as 6-deoxy-aginoside (formerly known also as F-gitonin) and 6-*epi*-aginoside, were isolated from *Allium cyrillii* [25] and from *Allium jesdianum* [26]. Aginoside and its 25*S* epimer, were identified in *Allium schubertii* bulbs [27]. Such close structural relationships of saponins in several related species of the genus *Allium* seems to indicate a species variability. Moreover, aginoside (**1**) and agigenin (**4**) were also isolated from our leek flower extract. This may indicate not only the known agronomic or climate variability [21] but also a possible organ variability. It may even denote a variability within leek varieties, as the leek is frequently cultivated in several varieties [12].

### 2.2. Biological Activities of Selected Saponins

**Figure 2:**
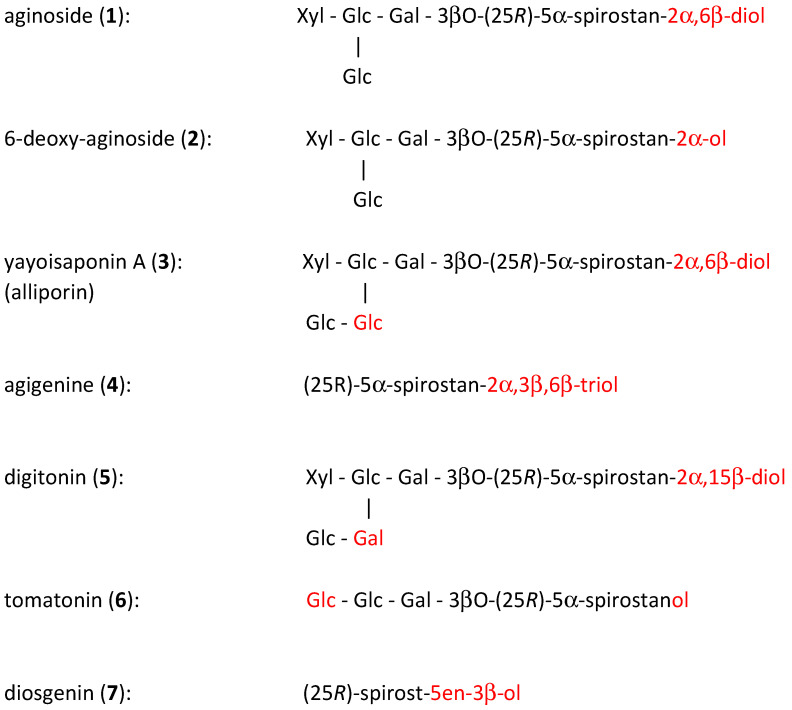
Schematic illustration of structural relations between the native leek-flower Compounds **1**–**4** and selected standard compounds **5**–**7**. Related saponins **5** and **6**, and aglycone **7** were selected for comparative bioactivity testing.

The isolated Compounds **1**–**4**, together with other structurally related saponins **5** and **6** and with the common spirostanol **7**, were selected for our cytotoxic and NO-production inhibitory activity bioassay. All tested saponins contain 3βO-bounded sugar moieties and possess 5αH configuration (i.e., *trans*-annelated A-B rings formation). The differences between the three native *Allium* saponins (**1**–**3**) consist only in the number of hydroxyls at position C-6 (in Compounds **1** and **2**) or in a prolonged chain of sugars in alliporin, now identified as yayoisaponin A (**3**), This is more distinctly illustrated in Figure 2.

Compounds **5**–**7** were involved in testing for a more extended structure-activity relationship evaluation. The well-known digitonin (**5**) was selected for comparing its activity with alliporin, i.e., yayoisaponin A (**3**) because of similarities in their structures (equal in numbers of hydroxyls and sugars), although there are some differences in their position (C-15 instead of C-6 for hydroxyls and Gal instead of Glc in the sugar sequence) (see Figure 2). Tomatonin (**6**) was selected because it lacks free hydroxyls at the aglycone part and contains a shorter glycosidic chain. Diosgenin (**7**) represents here a well-known bioactive steroid constituent structurally related to spirostanol sapogenins in the genus *Allium* [28], only lacking in its structure the C-2, C-6 or C-15 hydroxyls. Its Δ^5,6^ double bond affects only insignificantly the real A/B rings conformation.

### 2.3. In Vitro Biological Effects

**Figure 3:**
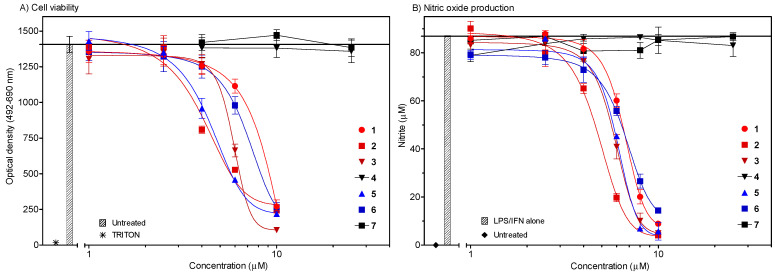
Cytotoxicity (**A**) and NO inhibitory effects (**B**) of Compounds **1**–**7** in mouse peritoneal cells. (**A**) Compounds were applied at appropriate concentrations and cells were cultured for 24 h. LDH assay was used for viability evaluation. The results are expressed in optical density of untreated control or treated cells ± SEM of *n* = 8 values from two independent experiments. (**B**) The cells were treated with compounds for 24 h with or without LPS (lipopolysaccharide) and IFN-γ (interferon-gamma). The results represent the mean ± SEM of two independent experiments, *n* = 6.

All sugars containing saponins (**1**, **2**, **3**, **5**, **6**) were found to possess strong cytotoxic effects in model immune cells (Figure 3A). The onset of cell viability decline was observed with the concentration of approximately 4 µM. A rapid decrease was reached with 10 µM concentrations, nearly at the bottom of the curve. In parallel, the same compounds inhibited the production of NO (Figure 3B).

Concentrations that required reducing the viability of cells and NO production by 50% (IC_50_, and CC_50_, respectively) were found to be very similar (see Table 1). A very tight correlation between these two parameters (r_/5/_ = 0.985, *p* < 0.01) suggests that cytotoxicity is a plausible explanation for the effects on NO production in mouse peritoneal macrophages. We used the LDH assay in our study. LDH and its release into the culture medium is an indicator of irreversible cell death due to cell membrane damage.

It is noteworthy that aglycon species **4** and **7** were found to be devoid of any inhibitory effects, although they were tested up to the relatively high concentration of 25 µM. The effect of these two compounds on cytotoxicity in immune cells is nearly missing. No changes in cytotoxicity were observed in RAW 246.7 macrophages and lymphocytes during the treatment with different concentrations of diosgenin (**7**) for 72 h [29]. In another study [30], agigenin (**4**) did not show cytotoxicity during 24 h in the murine monocyte/macrophage cell line J-774. Both studies, in which MTT assay was used for the measurement of cytotoxicity, support our findings on primary macrophages.

The exhibited toxicity data listed in Table 1, if compared with the cytotoxicity data recorded in the *Drosophila melanogaster* B_II_ cell bioassay [15,18], show noticeable similarities for the examined saponins in both systems, including inactivity for their aglycones. Another comparison can be observed with structurally related saponins from *Allium flavum* [31] evaluated for cytotoxicity against a human cancer cell line (colorectal SW480), showing only moderate cytotoxicity. Aginoside (**1**) isolated also from a mutant of elephant garlic *Allium ampeloprasum* [16] showed moderate cytotoxicity (IC_50_ = 2.1 µg/mL) in cancer murine leukemia cells P388 if compared to dioscin (IC_50_ = 0.092 µg/mL).

The remarkable similarity in the course of NO production values of all tested saponins **1**–**3**, **5** and **6** indicate that the structural differences in the sugars content, or in the number and position of hydroxyls in their aglycone part, play only a small role. Similarly, the structural differences of both tested aglycones **4** and **7** do not show apparent differences. Moreover, the course of changes in their NO production was almost zero in the range of the tested concentrations, similar to the activities of mutually related phyto-ecdysteroids [32] tested previously [8]. Various other activities of *Allium* saponins described in [1,2] are involved in various concepts [33,34], especially in the plant defence against insect pests [35,36,37] or against a series of fungi pathogens [38,39,40], confirming their relevant chemo-ecological role [3,12,15].

It seems that the potential immunobiological activity of our tested leek native saponins **1**–**3** is concealed probably only in a cumulative effect with their toxicity. This remains an interesting topic for further investigation, especially in the context of supporting their suggested immunological adjuvant activity [33,41], anti-inflammatory and anti-proliferative activity [42], cancer related and immunomodulatory activity [43,44], or their suitability for nutraceutical application [34,45].

Digitonin (**5**) is used as experimental tool for plasma membrane permeabilization. It is possible that structurally close spirostanol saponins, i.e., Compounds (**1**–**3**), possess digitonin-like effect and can thus represent an alternative source of natural compounds with specific properties. LDH toxicity assay belongs to one of basic test for monitoring time- and dose-dependent effects of drug treatment. At present, Dawid et al. [46] compared several pure saponins for respirometric assays in cell cultures. The authors found adequate alternative to digitonin to permeabilize the plasma membrane and superior to digitonin in tolerability for mitochondria. Importantly, LDH assay is applicable for various studies in cell cultures including 3D spheroids [47]. Besides that, other cytotoxic saponins isolated from flowers of endemic Caucasian *Allium leucanthum* [48] were found active for lung cancer cell line (A549) and colon cancer cell line (DLD-1).

## 3. Materials and Methods

### 3.1. General Methods

Melting points were determined on the Koffler block (Boetius) without correction. Optical rotations were measured using polarimeter Autopol IV (Rudolph Research Analytical, Flanders, NJ, USA). NMR spectra were measured on a Bruker AVANCE III HD 600 of Bruker Gmbh, Germany (^1^H at 600.13 MHz and ^13^C at 150.9 MHz) using a 5 mm TXI cryoprobe (Bruker Gmbh, Germany), in d_5_-pyridine at 25 °C. Chemical shifts were referenced to the solvent signal (δ_H(3,5)_ = 7.20, δ_C(4)_ = 135.5). The additional set of NMR spectra was measured for alliporin (**3**) in d_4_-methanol and chemical shift referenced to the solvent signal (δ_H_ = 3.31, δ_C_ = 49.0). Mass spectra, including HR-MS, were recorded on LTQ Orbitrap XL (Thermo Fisher Scientific, Bremen, Germany) spectrometer.

### 3.2. Chemicals

Aginoside **1** and its aglycone agigenin **4** had been obtained already in our earlier investigation [12,13]. They were again isolated in larger quantities from the stored fractions of the previous separation [13]. The minor constituents 6-deoxy-aginoside (**2**) and alliporin (**3**) were obtained by additional separation (see Section 3.4) from identical plant source and in the same procedure as before [15]. The compounds were identified by MS and NMR spectroscopy, and they were mutually compared with our original stored samples. Additional samples **5**–**7** (see Figure 2) were obtained from external sources. Prof. Kintia from the Academy of Sciences, Kishinev, Moldova [49] provided us with tomatonin (**5**). Digitonin (**6**) and diosgenin (**7**) were purchased from the Sigma-Aldrich Company and were purified by column chromatography [50].

### 3.3. Plant Material

The flowers of *Allium porrum* L. (cultivated leek “Malabare”) were obtained from the experimental fields of the Institut de Biocénotique Expérimentale des Agrosystèmes (IBEAS) Université François Rabelais, Tours, France. Leek flowers were dried immediately after their harvest (at 60 °C) and subsequently transported to our laboratory for further processing (see Section 3.4). Specimens were stored at IBEAS Tours.

### 3.4. Separation and Purification of Compounds

The compounds were extracted from the dried and powdered leek flowers (530 g) in a short-term percolation (2 h) with petroleum ether (Pe) for removing low-polar aliphatic (waxy and oily) substances (4.2 g). Repeated extraction with (2 × 2 L) ethyl acetate (EtOAc) followed for removing the next part of the low polar constituents (2.1 g). The next extraction, with (3 × 2 L) methanol (MeOH), provided a low molecular polar MeOH extract (98 g). The residue was subsequently extracted with MeOH-water (1:1), providing the extract (220 g) containing the expected saponin containing fraction, according to our previous experience [13]. After evaporating MeOH and part of the water (under reduced pressure), the remaining water part was extracted with (5 × 0.5 L) *n*-butanol (BuOH), providing a crude saponin fraction (29 g) without the undesirable ballast admixtures.

In the BuOH extract, aginoside (**1**) was detected by using an authentic sample from our earlier research [13] for monitoring and detection. In addition, it indicated also the presence of other saponins. The BuOH extract was fractionated by column chromatography on a silica gel (2 kg). For elution, the chloroform–MeOH-water (CHCl_3_-MeOH-H_2_O) solvent mixture was used with an increasing gradient of polar components (14:2:0–14:4:0–14:6:0–14:6:1). The process was monitored by TLC (CHCl_3_-MeOH-H_2_O = 14:6:1), and chromatographic fractions were distributed in combined fractions containing the single substances.

Compound **1** (208 mg) was obtained from relevant chromatographic fractions as white powder directly after evaporating the solvents. Compounds **2** (25 mg) and **3** (33 mg) were purified by repeated column chromatography of subsequent minor chromatographic fractions in the same solvent systems as indicated above. Compound **4** (12 mg) was isolated in a similar repeated column chromatography procedure by using solvent CHCl_3_-MeOH (20:1). It was detected also in the MeOH extract.

Compounds **5**–**7** were purified by flash chromatography on short silica gel columns using solvents: chloroform—methanol—water, 14:6:1. Purified compounds were subsequently inspected by HPLC analysis using Knauer-modular HPLC system equipped with reverse phase Separon SGX C-18 (7 μm) columns, produced by Tessek, Praha. The analysis was performed in a gradient mode with combining solvents (55–100% water in methanol), as reported in [51].

### 3.5. Identification of Compounds

The structure of each isolated compound was confirmed by ^1^H and ^13^C-NMR spectroscopy in d_5_-pyridine or CD_3_OD, supported by mass spectrometry analysis. Proton 1D- and homonuclear 2D-H,H-COSY, 2D-H,H-TOCSY, and 2D-H,H-ROESY spectra in combination with 1D-^13^C-APT and heteronuclear 2D-H,C-HSQC, and 2D-H,C-HMBC spectra were used for structural assignment of proton and carbon signals (see Table 2 and Table 3).

In this way, four structurally related compounds were identified: the already known aginoside (**1**) [13,17], its aglycone agigenin (**4**) [13], 6-deoxy-aginoside (**2**) [15,19], and a structurally related saponin named preliminarily alliporin (**3**) [3,15]. Compound **3** showed nearly identical chemical shifts of the aglycone part as aginoside (**1**) and a presence of additional hexapyranose (see MS data below).

NMR data of Compound **3** obtained using d_5_-pyridine as a solvent (see Table 2) indicated a possible structural identity with the previously reported yayoisaponin A [16] but with very few distinct differences in their data. Thus, the identity required a more reliable proof.

For our detailed structure analysis of Compound **3** we preferred to use NMR spectra in CD_3_OD (see Table 3) with removed OH signals and their *J*-couplings by deuterium exchange, thus affording a more advantageous and simplified approach to the analysis.

**Figure 4:**
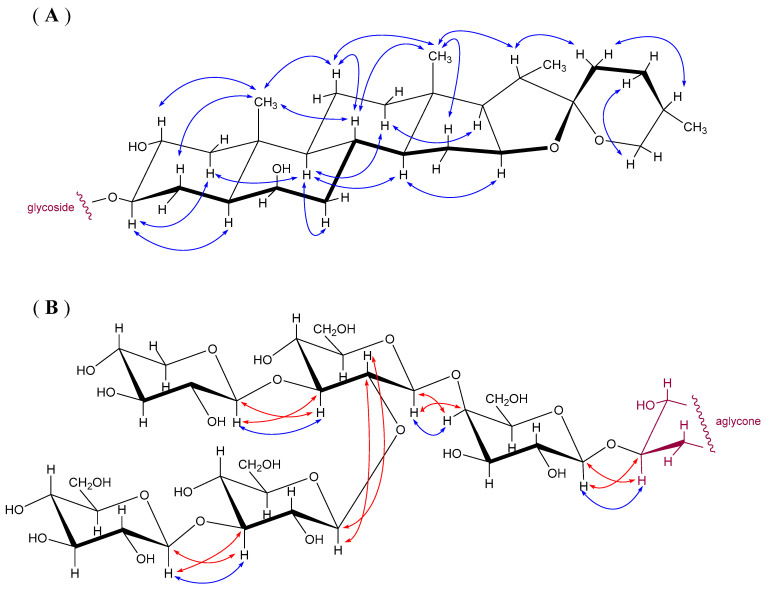
Selected NOE contacts (blue arrows) and ^3^*J*(C,H) (red arrows) observed in 2D-H,H-ROESY and 2D-H,C-HMBC spectra in CD_3_OD of alliporin, hereby identified as yayoisaponin A (**3**). (**A**) Aglycone: NOE contacts allow stereochemical assignment of methylene protons and prove the configuration at chiral centres. (**B**) Glycosidic part: NOE contacts and cross peaks in HMBC spectrum determining connection between hexapyranose units are shown. The ^13^C and ^1^H-NMR data are in Table 3.

The NOE contacts observed in the 2D-H,H-ROESY spectrum allowed a stereochemical assignment of geminal protons and proved configurations at the chiral centres of the aglycone part, as schematically shown in Figure 4A. Proton signals of individual hexopyranose units were assigned by 2D-H,H-COSY and 2D-H,H-TOCSY spectra. The mutual connection of sugar residues was determined from 2D-H,C-HMBC spectra (couplings between connected residues (i), (I + 1): *J* (H_(I + 1)_-C_(I + 1)_-O-C_(i)_) and *J* (C_(I + 1)_-O-C_(i)_-H_(i)_)) and NOE contacts as shown in Figure 4B. The set of NMR experiments was extended with homonuclear 2D-*J*-resolved spectrum that proved to be very helpful in the identification of proton multiplets (often partly overlapped in 1D proton spectra) and for the determination of *J*(H,H) values. Thus, four monosaccharide units were shown identical to the units in aginoside (**1**). The fifth unit was identified as β-D-glucopyranose connected to position 3 of Glc(IV) in Compound **3**, as observed in yayoisaponin A [16] or as indicated in our previous communications under the name alliporin [3,15]. The structure analysis illustrated in Figure 4 is based on NMR data measured in CD_3_OD, summarized in Table 3. It confirms the identity of alliporin with yayoisaponin A (**3**).

#### 3.5.1. Aginoside (**1**)

White amorphous powder with m. p. 250–252 °C and [α]_D_ −53.1 °C (c 0.51 in CHCl_3_-CH_3_OH 1:1). FTMS + p ESI: composition C_50_H_82_O_24_ (M = 1066) determined by HR-MS: 1089.50884 [M + Na], for C_50_H_82_O_24_Na calculated 1089.50882. ^1^H and ^13^C-NMR data are in Table 2.

#### 3.5.2. 6-Deoxy-Aginoside (**2**)

White amorphous powder. [α]_D_ −53.9 °C (c 0.17 in CHCl_3_-CH_3_OH 1:1). FTMS + p ESI: Composition C_50_H_82_O_23_ (M = 1050) determined by HR-MS: 1073.51393 [M + Na], for C_50_H_82_O_23_Na calculated 1073.51391. ^1^H and ^13^C-NMR data are in Table 2.

#### 3.5.3. Yayoisaponin A (**3**), Previously Known also as Alliporin

White amorphous powder. [α]_D_ −45.1 °C (c 0.15 in CHCl_3_-CH_3_OH 1:1). FTMS + p ESI: Composition C_56_H_92_O_29_ (M = 1228) determined by HR-MS: 1251.56226 [M + Na], for C_56_H_92_O_29_Na calculated 1251.56165. ^1^H and ^13^C-NMR data are in Table 2 and Table 3.

#### 3.5.4. Agigenin (**4**)

White amorphous powder with m. p. 271–273 °C and [α]_D_−54.4 °C (c 0.13 in CHCl_3_). FTMS + p ESI: Composition C_27_H_44_O_5_ (M = 448) determined by HR-MS: 471.30817 [M + Na], for C_27_H_44_O_5_Na calculated 471.30810. ^1^H and ^13^C-NMR data are in Table 2.

### 3.6. Biological Assays

Biological effects of studied compounds were evaluated in vitro, using mouse (C57BL6, Charles River Deutschland, Sulzfeld, Germany) peritoneal cells. Animals, killed by cervical dislocation, were i.p. injected with 8 mL of sterile saline. Pooled peritoneal cells collected from mice (*n* = 4–6 in individual experiments) were washed, re-suspended in culture medium, and seeded into 96-well round-bottom microplates (Costar, Corning, NY, USA) in 100-μL volumes, 2 × 10^5^ cells/well. All experimental variants were run in duplicate. Complete RPMI-1640 culture medium (Sigma-Aldrich, St. Louis, MO, USA) contained 10% heat-inactivated foetal bovine serum, 2 mM L-glutamine, 50 μg/mL gentamicin, and 5 × 10^−5^ M 2-mercaptoethanol (all Sigma-Aldrich). Cultures were maintained at 37 °C, 5% CO_2_ in humidified incubator (Sanyo Electric Biomedical, Osaka, Japan). The Institution Animal Ethics Committee (No. 13/2006) approved the animal welfare and all experimental procedures.

#### 3.6.1. Nitric Oxide (NO) Production

High output NO production was induced by mixture of lipopolysaccharide (LPS from *E. coli* 0111:B4, 0.1 ng/mL; Sigma) and murine recombinant interferon-γ (IFN-γ, 5 ng/mL; R&D Systems, Minneapolis, MN, USA) in mouse peritoneal cells. Tested compounds were applied concomitantly with these priming stimuli. The concentration of nitrites in supernatants of cells was assayed at the interval of 24 h. It was detected in individual, cell-free samples (50 μL) incubated 5 min at ambient temperature with an aliquot of a Griess reagent (1% sulphanilamide/0.1% naphtylendiamine/2.5% H_3_PO_4_). The absorbance at 540 nm was recorded using a microplate spectrophotometer (Tecan, Grödig, Austria). A nitrite calibration curve was used to convert absorbance to μM nitrite.

#### 3.6.2. Cell Viability

Viability of cells was analysed using the LDH (lactate dehydrogenase) assay. It is based on the determination of lactate dehydrogenase activity released from the cytosol of damaged cells into cell supernatant. The supernatants were harvested at the interval of 22 h of culture, diluted 1:1, and mixed with an aliquot of the LDH kit (Sigma-Aldrich, St. Louis, MO, USA). After 30-min incubation in the dark at ambient temperature, the reaction was stopped with 2 N HCl. Differences between the absorbance at 492–690 nm were evaluated. Triton (1%) was used to induce 100% cell death. All control and experimental variants were run in quadruplicate. Similar methodology for LDH toxicity assay is applied in a related treatment, including macrophages [52].

#### 3.6.3. Statistical Analysis

Estimates of 50% inhibitory concentrations of compounds (IC_50_, and CC_50_), correlation analysis, and graphical presentation of data were done using the Prism program (GraphPad Software, San Diego, CA, USA).

## 4. Conclusions

Three structurally related spirostanol saponins **1**–**3** were isolated from leek flowers and structurally identified by MS and NMR analysis. Yayoisaponin A (**3**) is a new compound found in *Allium porrum*, although it was already known in another species of the genus *Allium*. Leek flower saponins **1**–**3** were tested together with other structurally related spirostanol Compounds **4**–**7** for in vitro cytotoxicity and for effects on NO production. The obtained toxicity data closely correlated with the suppression of NO production. The highest inhibitory effects on viability (LDH assay) were exhibited by 6-deoxyaginoside (**2**), which is comparable to the well-known digitonin (**5**). However, the activity differences between all tested saponins are negligible.

Immune cells play an important role in health and diseases. Our results showed for the first time cytotoxicity/viability effects of spirostanol saponins on rodent peritoneal cells. The pilot results based on LDH assays should be supplemented by additional viability tests and culture models to explain biological activities of saponins and their rational applications for human in biomedicine.

An important fact should be noted: the quantitative content of saponins in the leek flowers is 20 times higher than in the edible part of the plant [3,13], so leek flowers can be considered as a new economically advantageous source of saponins. Flowers may even have a different chemical composition than other parts of the plant [53]. In our case, it is only about quantitative differences that play a role. Since leeks are widely grown in agriculture, the respective saponins can be gained advantageously without any extra investment.

## Figures and Tables

**Table 1:** Concentrations required for reducing the viability of cells and NO production by 50% (CC_50_ and IC_50_, respectively).

Compound	Cytotoxicity CC_50_ (µM) (95% Limits of Confidence)	Nitric Oxide, IC_50_ (µM) (95% Limits of Confidence)
**1**. aginoside	11.13 (2.76–44.91)	7.84 (2.16–28.51)
**2**. 6-deoxy-aginoside	5.70 (1.83–17.71)	5.21 (1.17–23.14)
**3**. yayoisaponin A (alliporin)	7.21 (1.64–31.47)	5.62 (1.59–19.24)
**4**. agigenin	>100	>100
**5**. digitonin	5.83 (1.72–19.75)	5.52 (1.57–19.46)
**6**. tomatonin	9.90 (2.80–35.03)	7.19 (2.99–17.29)
**7**. diosgenin	>100	>100

**Table 2:** ^13^C and ^1^H NMR chemical shifts of compounds **1**–**4** in d_5_-pyridine *^a^*.

Position	Aginoside (1)		6-Deoxyaginoside (2)		Alliporin (3)		Agigenin (4)	
Aglycone	^13^C	^1^H	^13^C	^1^H	^13^C	^1^H	^13^C	^1^H
1	46.94	1.22; 2.18	45.38	1.12; 2.15	46.89	1.20; 2.17	48.10	1.37; 2.28
2	70.36	4.07	70.28	3.93	70.35	4.07	73.21	4.19
3	84.36	4.01	84.01	3.85	84.42	4.00	77.33	3.97
4	31.72	2.12; 2.36	33.83	1.43; 1.82	31.60	2.12; 2.36	35.10	2.10; 2.53
5	47.64	1.13	44.38	0.96	47.60	1.12	48.49	1.40
6	69.80	3.96	27.90	0.98; 1.11	69.79	3.95	70.31	4.09
7	40.67	1.13; 1.99	31.93	*^b^* ; 1.45	40.65	1.12; 1.98	40.88	1.21; 2.04
8	29.83	2.14	34.37	1.33	29.80	2.13	30.08	2.23
9	54.32	0.71	54.15	0.53	54.30	0.69	54.75	0.83
10	36.84	--	36.67	--	36.81	--	37.71	--
11	21.16	1.35; 1.52	21.23	*^b^* ; 1.40	21.16	1.35; 1.50	21.46	1.47; 1.64
12	39.90	1.04; 1.65	39.86	0.96; 1.59	39.90	1.05; 1.64	40.20	1.14; 1.71
13	40.50	--	40.59	--	40.47	--	40.93	--
14	56.03	1.10	56.13	0.98	56.02	1.08	56.29	1.16
15	32.04	1.40; 2.05	32.00	1.36; 1.98	32.03	1.40; 2.05	32.24	1.44; 2.10
16	80.95	4.55	80.98	4.53	80.94	4.53	81.11	4.56
17	62.82	1.81	62.79	1.76	62.79	1.80	63.04	1.85
18	16.40	0.82	16.42	0.77	16.38	0.81	16.62	0.87
19	17.01	1.25	13.22	0.66	16.98	1.24	17.55	1.44
20	41.82	1.91	41.80	1.91	41.79	1.90	41.99	1.95
21	14.83	1.12	14.83	1.10	14.82	1.10	15.03	1.14
22	109.09	--	109.12	--	109.08	--	109.21	--
23	31.58	1.56; 1.63	31.62	1.63 (2H)	31.55	1.57; 1.63	31.74	1.60; 1.67
24	29.04	1.24; 1.54	29.08	1.23; 1.53	29.01	1.50 (2H)	29.22	1.53 (2H)
25	30.39	1.54	30.41	1.54	30.37	1.52	30.58	1.55
26	66.68	3.47; 3.57	66.70	3.48; 3.57	66.66	3.45; 3.55	66.83	3.49; 3.57
27	17.12	0.66	17.15	0.67	17.11	0.64	17.30	0.67
Gal (I)								
1	102.92	4.96	103.08	4.90	102.87	4.94		
2	72.35	4.54	72.41	4.51	72.34	4.50		
3	75.57	4.03	75.58	4.03	75.54	4.02		
4	79.22	4.58	79.23	4.57	78.99	4.56		
5	75.36	4.12	75.35	4.11	75.39	4.12		
6	60.51	4.21; 4.59	60.50	4.20; 4.59	60.55	4.20; 4.57		
Glc (II)								
1	104.45	5.19	104.52	5.18	104.66	5.13		
2	81.05	4.33	81.06	4.33	80.94	4.53		
3	86.83	4.11	86.81	4.11	86.89	4.06		
4	70.19	3.77	70.22	3.78	70.11	3.76		
5	77.40	3.83	77.42	3.82	77.33	3.80		
6	62.72	4.04; 4.47	62.74	4.03; 4.47	62.65	4.04; 4.45		
Xyl (III)								
1	104.76	5.23	104.78	5.23	104.11	5.16		
2	74.94	3.94	74.96	3.94	74.99	4.05		
3	78.27	3.90	78.30	3.90	78.23	3.84		
4	70.60	4.10	70.62	4.09	70.47	4.07		
5	67.12	3.65; 4.20	67.14	3.64; 4.20	67.01	3.62; 4.17		
Glc (IV)								
1	104.59	5.57	104.62	5.57	103.68	5.57		
2	75.87	4.04	75.92	4.03	75.28	3.92		
3	77.98	4.14	77.98	4.13	87.70	4.10		
4	71.20	4.04	71.20	4.08	69.54	3.86		
5	78.50	4.07	78.52	4.07	77.83	4.12		
6	62.54	4.40; 4.53	62.53	4.39; 4.54	62.26	4.20; 4.45		
Glc (V)								
1					105.22	5.08		
2					77.56	3.80		
3					78.19	4.05		
4					71.33	4.09		
5					80.39	4.28		
6					62.20	4.25; 4.40		

*^a^* Chemical shifts were referenced to the solvent signal (δ_H(3,5)_ = 7.20; δ_C(4)_ = 135.5), *^b^* not determined value.

**Table 3:** ^13^C and ^1^H NMR data of alliporin (**3**) in CD_3_OD.

Aglycone			Glycosidic Part		
Position	^13^C	^1^H		^13^C	^1^H
1	47.14	H-1α: 0.93; H-1β: 1.905	Gal (I)		
2	71.34	3.67	1	102.84	4.385 d (*J* = 7.8)
3	85.02	3.56	2	72.86	3.72 dd (*J* = 9.7; 7.8)
4	31.47	H-4α: 1.755; H-4β: 1.84	3	75.50	3.55 dd (*J* = 9.7; 3.3)
5	48.46	1.205	4	79.94	4.05 dd (*J* = 3.3; 1.0)
6	71.52	3.805	5	75.67	3.56 ddd (7.4; 6.3; 1.0)
7	40.71	H-7α: 1.185; H-4β: 1.83	6	61.40	3.86 dd (*J* = 11.3; 7.4); 3.675 (*J* = 11.3; 6.3)
8	30.74	1.95	Glc (II)		
9	55.52	0.79	1	104.41	4.63 d (*J* = 7.6)
10	37.77	--	2	80.80	3.76 dd (*J* = 9.8; 7.6)
11	22.13	H-11α: 1.57; H-11β: 1.44	3	87.59	3.74 dd (*J* = 9.8; 8.2)
12	41.02	H-12α: 1.185; H-12β: 1.76	4	70.42	3.30 dd (*J* = 8.2; 9.8)
13	41.79	--	5	70.25	3.46 ddd (9.8; 7.0; 2.3)
14	57.13	1.18	6	63.04	3.90 dd (*J* = 11.6; 2.3); 3.595 dd (*J* = 11.6; 7.0)
15	32.70	H-15α: 2.00; H-15β: 1.30	Xyl (III)		
16	82.18	4.39	1	104.88	4.64 d (*J* = 7.7)
17	63.83	1.76	2	75.31	3.26 dd (*J* = 9.6; 7.7)
18	16.98	0.829	3	75.47	3.29 dd (*J* = 9.6; 8.0)
19	17.24	1.073	4	70.96	3.53 ddd (*J* = 10.3; 8.0; 5.9)
20	42.94	1.91	5	67.19	3.92 dd (*J* = 11.6; 5.9); 3.265 dd (11.6; 10.3)
21	14.88	0.963	Glc (IV)		
22	110.56	--	1	103.70	5.01 d (*J* = 8.0)
23	32.41	H-23α: 1.70; H-23β: 1.57	2	75.01	3.42 dd (*J* = 9.4; 8.0)
24	29.88	H-24α: 1.43; H-24β: 1.63	3	87.66	3.58 dd (*J* = 9.4; 8.3)
25	31.44	1.60	4	71.57	3.29 dd (*J* = 8.3; 9.7)
26	67.85	H-26α: 3.32; H-26β:3.45	5	78.10	3.34 ddd (*J* = 9.7; 6.3; 2.0)
27	17.49	0.794	6	62.87	3.92 (*J* = 12.2; 2.0); 3.83 dd (*J* = 12.2; 6.3)
			Glc (V)		
			1	105.21	4.57 d (*J* = 7.8)
			2	77.51	3.34 dd (*J* = 9.0; 7.8)
			3	77.78	3.40 t (*J* ~ 9.0; 9.0)
			4	77.96	3.345 dd (~9.0; 9.0)
			5	78.22	3.315 ddd (*J* ~9.0; 6.3; 2.3)
			6	62.63	3.89 dd (*J* = 11.8; 2.3); 3.645 dd (*J* = 11.8; 6.3)

Chemical shift referenced to the solvent signal (δ_H_ = 3.31, δ_C_ = 49.0).

## Data Availability

Not available.

## Sample Availability

Samples of the compounds **1**–**4** are available from the authors.

## References

[1] Hostettmann K., Marston A. (1995). Book, Saponins. Chemistry and Pharmacology of Natural Products.

[2] Waller G.R., Yamasaki K. (1996). Saponins Used in Traditional and Modern Medicine. Advances in Experimental Medicine and Biology.

[3] Harmatha J., Oleszek W., Marston A. (2000). Chemo-ecological role of spirostanol saponins in the interaction between plants and insects. Book Saponins in Food, Feedstuffs and Medicinal Plants.

[4] Lanzotti V. (2005). Bioactive saponins from *Allium* and *Aster* plants. Phytochem. Rev..

[5] Lanzotti V. (2012). Bioactive polar natural compounds from garlic and onions. Phytochem. Rev..

[6] Sobolewska D., Michalska K., Podolak I., Grabowska K. (2016). Steroidal saponins from the genus *Allium*. Phytochem. Rev..

[7] Francis G., Kerem Z., Makkar H.P.S., Becker K. (2002). The biological action of saponins in animal systems: A review. Br. J. Nutr..

[8] Harmatha J., Vokáč K., Kmoníčková E., Zídek Z. (2008). Lack of interference of common phytoecdysteroids with production of nitric oxide by immune-activated mammalian macrophages. Steroids.

[9] Harmatha J., Buděšínský M., Vokáč K., Kostecká P., Kmoníčková E., Zídek Z. (2013). Trilobolide and related sesquiterpene lactones from *Laser trilobum* possessing immunobiological properties. Fitoterapia.

[10] Harmatha J., Vokáč K., Buděšínský M., Zídek Z., Kmoníčková E. (2015). Immunobiological properties of sesquiterpene lactones obtained by chemically transformed structural modifications of trilobolide. Fitoterapia.

[11] Harmatha J., Buděšínský M., Jurášek M., Zimmermann T., Drašar P., Zídek Z., Kmoníčková E., Vejvodová L. (2019). Structural modification of trilobolide for upgrading its immunobiological properties and reducing its cytotoxic action. Fitoterapia.

[12] Arnault C., Harmatha J., Mauchamp B., Sláma K., Labeyrie V., Fabres G., Lachaise D. (1987). Influence of allelochemical substances of the host plant (*Allium porrum*) on development and moulting of *Acrolepiopsis assectella* (Lepidoptera). Their Role as Selective Factor.

[13] Harmatha J., Mauchamp B., Arnault C., Sláma K. (1987). Identification of a spirostane-type saponin in the flowers of leek with inhibitory effects on growth of leek-moth larvae. Biochem. Syst. Ecol..

[14] Arnault C., Mauchamp B. (1985). Ecdysis inhibition in *Acrolepiopsis assectella* larvae by digitoxin: Antagonistic effects of cholesterol. Experientia.

[15] Harmatha J., Dinan L., Konopinska D. (2002). Interaction of dimeric ecdysteroids, glycosidic ecdysteroid conjugates and ecdysis-disturbing saponins with the ecdysteroid receptor assessed by means of the *Drosophila melanogaster* B-II bioassay. Book Arthropods: Chemical, Physiological and Environmental Aspects.

[16] Sata N., Matsunaga S., Fusetani N., Nushikawa H., Takamura S., Saito T. (1998). New antifungal and cytotoxic steroidal saponins from the bulbs of elephant garlic mutant. Biosci. Biotechnol. Biochem..

[17] Kelginbayev A.N., Gorovits M.B., Gorovits T.T., Abubakirov N.K. (1976). *Allium* steroid saponins and sapogenins IX—Structure of aginosid. Khim. Prir. Soedin..

[18] Dinan L., Bourne P.C., Meng Y., Sarker S.D., Tolentino R.B. (2001). Whiting, Assessment of natural products in the *Drosophila melanogaster* BII cell bioassay for ecdysteroid agonist and antagonist activities. CMLS Cell. Mol. Life Sci..

[19] Carotenuto A., Fattorusso E., Lanzotti V., Magno S. (1999). Spirostanol saponins of *Allium porrum* L.. Phytochemistry.

[20] Fattorusso E., Lanzotti V., Taglialatela-Scafati O., Di Rosa M., Ianaro A. (2000). Cytotoxic saponins from bulbs of *Allium porrum* L.. J. Agric. Food Chem..

[21] Fattorusso E., Lanzotti V., Magno S., Taglialatela-Scafati O. (1998). Sapogenins of *Allium porrum* L.. J. Agric. Food Chem..

[22] Gvazava L.N., Skhirtladze A.V. (2017). Steroidal saponin from *Allium porrum*. Chem. Nat. Comp..

[23] Gvazava L.N., Skhirtladze A.V. (2018). Steroidal glycoside from *Allium porrum*. Chem. Nat. Comp..

[24] Maisashvili M.R., Kuchukhidze D.K., Kikoladze V.S., Gvazava L.N. (2012). Steroidal glycosides of gitogenin from *Allium rotundum*. Chem. Nat. Comp..

[25] Tolkacheva N.V., Shashkov A.S., Chirva V.Y. (2012). Steroidal glycosides from *Allium cyrillii* bulbs. Chem. Nat. Comp..

[26] Mimaki Y., Kuroda M., Fukasawa T., Sashida Y. (1999). Steroidal glycosides from bulbs of *Allium jesdianum*. J. Nat. Prod..

[27] Kawashima K., Minaki Y., Sashida Y. (1993). Steroidal saponins from the bulbs of *Allium schubertii*. Phytochemistry.

[28] Kravets S.D., Vollerner Y.S., Gorovits M.B., Abubakirov N.K. (1990). Steroids of the spirostan and furostan series from plants of the genus *Allium*. Khim. Prir. Soed..

[29] Selim S., Al Jaouni S. (2015). Anticancer and apoptotic effects on cell proliferation of diosgenin isolated from *Costus speciosus* (Koen.) Sm. BMC Complemet. Altern. Med..

[30] Carotenuto A., Fattorusso E., Lanzotti V., Magno S., De Feo V., Carnuccio R., D’Acquisto F. (1997). Porrigenins A and B, Novel Cytotoxic and Antiproliferative Sapogenins Isolated from *Allium porrum*. J. Nat. Prod..

[31] Rezgui A., Mitaine-Offer A.C., Paululat T., Delemasure S., Dutartre P., Lacaille-Dubois M.-A. (2014). Cytotoxic steroidal glycosides from *Allium flavum*. Fitoterapia.

[32] Lafont R., Harmatha J., Marion-Poll F., Dinan L., Wilson I.D. (2002). The Ecdysone Handbook.

[33] Rodrigues Adao C., Pereira da Silva B., Wanderley Tinoco L., Paz Parente J. (2012). Haemolytic Activity and Immunological Adjuvant Effect of a New Steroidal Saponin from *Allium ampeloprasum* var. porrum. Chem. Biodivers..

[34] Nasri H., Baradaran A., Shirzad H., Rafieian-Kopaei M. (2014). New concepts in nutraceuticals as alternative for pharmaceuticals. Int. J. Prev. Med..

[35] De Geyter E., Lambert E., Geelen D., Smagghe G. (2007). Novel advances with plant saponins as natural insecticides to control pest insects. Pest Technol..

[36] Chaieb I. (2010). Saponins as Insecticides: A Review. Tunisian J. Plant Protect..

[37] Singh B., Kaur A. (2018). Control of insect pests in crop plants and stored food grains using plant saponins: A review. Food Sci. Technol..

[38] Barile E., Bonanomi G., Antignani V., Zolfaghari B., Sajjadi S.E., Scala F., Lanzotti V. (2007). Saponins from *Allium minutiflorum* with antifungal activity. Phytochemistry.

[39] Lanzotti V., Barile E., Antignani V., Bonanomi G., Scala F. (2012). Antifungal saponins from bulbs of garlic, *Allium sativum* L. var. Voghiera. Phytochemistry.

[40] Mostafa A., Sudisha J., El-Sayed M., Ito S., Ikeda T., Yamauchi N., Shigyo M. (2013). Aginoside saponin, a potent antifungal compound, and secondary metabolite analyses from *Allium nigrum* L.. Phytochem. Lett..

[41] Sparg S.G., Light M.E., van Staden J. (2004). Biological activities and distribution of plant saponins. J. Ethnopharm..

[42] Wang Y., Li C., Xiang L., Huang W., He X. (2016). Spirostanol saponins from Chinese onion (*Allium chinense*) exert pronounced anti-inflammatory and anti-proliferative activities. J. Funcion. Foods.

[43] Lacaille-Dubois M.-A. (2005). Bioactive saponins with cancer related and immunomodulatory activity: Recent developments. Stud. Nat. Prod. Chem. (Part L).

[44] Jabrane A., Ben Jannet H., Miyamoto T., Mirjolet J.-F., Duchamp O., Harzallah-Skhiri F., Lacaille-Dubois M.A. (2011). Spirostane and cholestane glycosides from the bulbs of *Allium nigrum* L.. Food Chem..

[45] Upadhyay R.K. (2017). Nutritional and therapeutic potential of *Allium* vegetables. J. Nutr. Therap..

[46] Dawid C., Weber D., Musiol E., Janas V., Baur S., Lang R., Fromme T. (2020). Comparative assessment of purified saponins as permeabilization agents during respiratory. Biochim. Biophys. Acta Bioenerg..

[47] Karassina N., Hofsteen P., Cali J.J., Vidugiriene J. (2021). Time- and dose-depentendt toxicity studies in 3D cultures using a luminiscent lactate dehydrogenase assay. Methods Mol. Biol..

[48] Mskhiladze L., Legault J., Lavoie S., Mshvildadze V., Kuchukhidze J., Elias R., Pichette A. (2008). Cytotoxic steroidal saponins from the flowers of *Allium leucanthum*. Molecules.

[49] Kintia P.K., Waller G.R., Yamasaki K. (1996). Chemistry and Biological Activity of Steroid Saponins from Moldavian Plants. Saponins Used in Traditional and Modern Medicine. Advances in Experimental Medicine and Biology.

[50] Dinan L., Harmatha J., Lafont R. (2001). Chromatographic procedures for the isolation of plant steroids. J. Chromatogr. A.

[51] Dinan L., Harmatha J., Lafont R., Waksmundzka-Hajnos M., Sherma J. (2011). HPLC of Steroids. High Performance Liquid Chromatography in Phytochemical Analysis.

[52] Vennemann A., Alessandrini F., Wiemann M. (2017). Differential Effects of Surface-Functionalized Zirconium Oxide Nanoparticles on Alveolar Macrophages, Rat Lung, and a Mouse Allergy Model. Nanomaterials.

[53] Gupta A.K., Rather M.A., Jha A.K., Shashank A., Singhal S., Sharma M., Pathak U., Sharma D., Mastinu A. (2020). *Artocarpus lakoocha* Roxb. and *Artocarpus heterophyllus* Lam. Flowers: New Sources of Bioactive Compounds. Plants.

